# MR2938 relieves DSS-induced colitis in mice through inhibiting NF-κB signaling and improving epithelial barrier

**DOI:** 10.1007/s42995-025-00285-x

**Published:** 2025-03-17

**Authors:** Ling Lv, Mireguli Maimaitiming, Shuli Xia, Jichen Yang, Tiantian Zhang, Yuming Wang, Xin Li, Iryna Pinchuk, Pingyuan Wang, Chang-Yun Wang, Zhiqing Liu

**Affiliations:** 1 Key Laboratory of Marine Drugs and Key Laboratory of Evolution and Marine Biodiversity (Ministry of Education), School of Medicine and Pharmacy, Institute of Evolution & Marine Biodiversity, College of Food Science and Engineering, Ocean University of China, Qingdao, 266003 China; 2 Laboratory for Marine Drugs and Bioproducts, Qingdao Marine Science and Technology Center, Qingdao, 266237 China; 3 Division of Gastroenterology, Department of Medicine, Pennsylvania State Milton S. Hershey Medical Center, Hershey, PA 17033 USA

**Keywords:** Inflammatory bowel diseases, Inflammation, NF-κB signaling, Barrier function

## Abstract

Damage to the epithelial barrier is among key processes contributing to initiation and chronic inflammation in inflammatory bowel diseases (IBD). Only management therapy exists for IBD (e.g., anti-inflammatory and immunomodulatory agents, JAK/STAT inhibitors), and while novel therapeutic approaches have shown great potential, issues remain including route of administration, development of resistance to therapy and toxicity. Thus, novel small molecule inhibitors which can alleviate colonic inflammation and restore intestinal barrier functions are needed. Our previous study identified a new quinazolinone derivative MR2938, inspired by marine natural product penipanoid C, displaying impressive anti-inflammatory effects. In vivo efficacy study indicated that MR2938 had a dose-dependent effect on improving colitis symptoms, gut-barrier disruption, and colonic inflammation in an acute dextran sulfate sodium (DSS)-induced murine colitis as a model of epithelial injury relevant to IBD. Evaluation of potential mechanism involved in MR2938 efficacy demonstrated that MR2938 inhibited NF-κB-mediated inflammatory responses, and attenuated intestinal epithelial tight junction damage by restoring the expression of Occludin and ZO-1. Taken together, these data suggest that MR2938 is a promising lead compound for the treatment of IBD.

**Supplementary Information:**

The online version contains supplementary material available at 10.1007/s42995-025-00285-x.

## Introduction

**Fig. 1:**
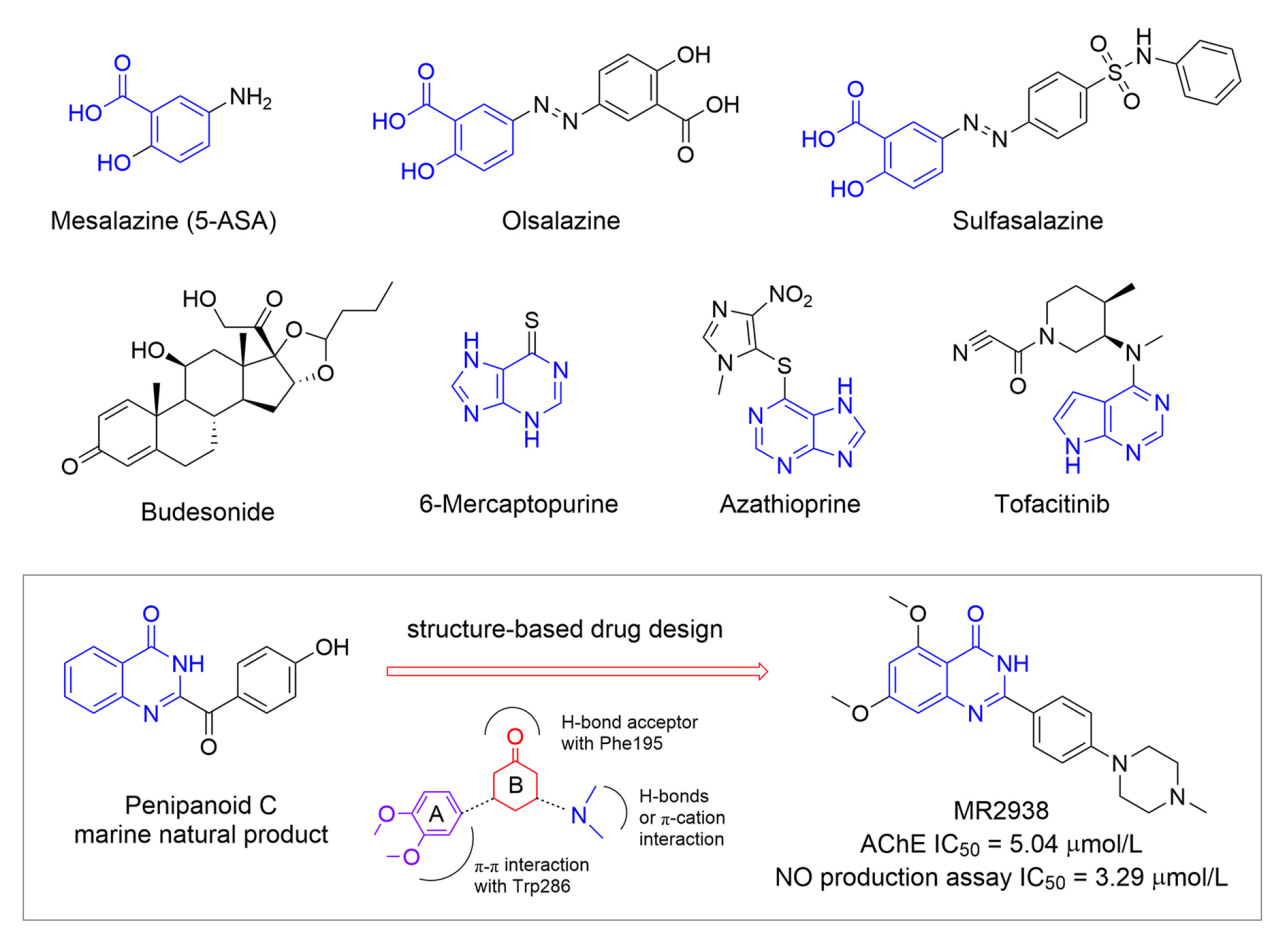
Representative FDA-approved drugs for IBD and discovery of MR2938

Inflammatory bowel disease (IBD) affects about 7 million people globally, and the number is increasing worldwide (Burisch et al. 2023; Le Berre et al. 2023). Crohn’s disease (CD) and ulcerative colitis (UC) are two major forms of chronic IBD and have increased by ~ 40% over the past 2 decades (Park et al. 2023). IBD is a multifactorial disease involving an abnormal immune response to gut microbiota in genetically susceptible individuals, resulting in chronic inflammation and epithelial dysfunction (Kobayashi et al. 2020; Kudelka et al. 2020). IBD is considered life-long disease because of high relapse rate, and long-term persistence of chronic inflammation may result in the development of colitis-associated colorectal cancer which may cause 10% to 15% mortality rate. The first-line treatment for mild to moderate IBD is 5-aminosalicylate (5-ASA) in different forms (e.g., Mesalamine, Olsalazine, and Sulfasalazine in Fig. 1) which decreases inflammation (Le Berre et al. 2020). For moderate and severe IBD patients, corticosteroids like budesonide can be used, but just for short term due to their serious side effects. Immunomodulators like thiopurines (e.g., 6-mercaptopurine and Azathioprine) work by suppressing inflammation and are representatives for second-line medications. Anti-TNF biologics were also able to reduce symptoms and maintain remission, but a large proportion of patients are unresponsive (Ito et al. 2004). Most new (e.g., Janus kinase inhibitor Tofacitinib) and emerging therapeutics (e.g., sphingosine 1-phosphate receptor modulator) are focused on maintaining the remission of IBD, and very few of them can restore mucosal damage and dysregulated intestinal barrier functions which is an initial event in the disease suppression and progression (Kotla et al. 2022). Further, there is an urgent need for the development of an orally available drug for IBD (Teruel et al. 2020; Zhang et al. 2021).

A complex interplay between intestinal mucosal dysfunction and dysregulated immune responses, mediated by intestinal epithelial and immune cells, allows the development of IBD (Chen et al. 2023; Li et al. 2023; Liyanage et al. 2023). Tight junctions are formed by intracellular linker proteins like ZO-1 interacting with transmembrane molecules including claudin, JAMs, and Occludin (Haas et al. 2020; Kuo et al. 2022). Together, tight junctions and adhesion junctions create a physical barrier, reinforced by the mucus secretion from goblet cells. This barrier effectively partitions the epithelial layer from the luminal microbiota, highlighting epithelial cells as pivotal in safeguarding the intestinal barrier (Furuse 2010, Gustafsson et al. 2022). The intricate immune system strengthens the intestinal barrier and triggers a strong immune response when the intestinal barrier is disrupted. Elevated levels of proinflammatory cytokines, such as IL-1β and TNF-α, not only dysregulated inflammatory responses in IBD patients but also have been shown to disrupt tight junctions and increase intestinal permeability (Al-Sadi and Ma 2007; Song et al. 2022). Thus, we believe small molecules that can suppress inflammation and improve intestinal barrier function at the same time may be more effective therapeutics for IBD.

Marine natural products (MNPs) and their derivatives represent a significant reservoir of biologically active compounds, serving as crucial lead structures for the development of therapeutic agents against cancer and inflammatory disorders. In our pursuit of MNPs as useful lead compounds or therapeutics for the treatment of inflammatory diseases, a series of quinazolin-4(3*H*)-one derivatives, inspired by MNP penipanoid C, have been designed and synthesized as novel multifunctional anti-AD agents demonstrating both cholinesterase inhibition and anti-inflammatory activities (Lv et al. 2023). Among them, MR2938 displayed promising AChE inhibitory activity with an IC_50_ value of 5.04 μmol/L and significantly decreased the mRNA level of proinflammatory cytokines at 2.5 μmol/L (Fig. 1). Recently we evaluated the pharmacokinetic (PK) profile of MR2938 in rats (Supporting Information Table S1), and brain permeability in mice. In rats, MR2938 displayed a reasonable half-life (T_1/2_ = 2.25 h and 2.87 h, respectively) and plasma exposure (AUC values of 1,071 h·ng/mL and 2,107 h·ng/mL, respectively) via i.v. (3 mg/kg) and p.o. (10 mg/kg) administration. Overall, MR2938 demonstrated good oral bioavailability with *F* value of 59%. Notably, MR2938 may not be suitable for neurological disorders, as it can hardly penetrate blood–brain barrier with a brain/plasma ratio of 0.05% (Table S2). Thus, considering the promising anti-inflammatory activity and druggability of MR2938, we hypothesize that it may be an orally available and effective lead compound for IBD.

To validate this hypothesis, we investigated the therapeutic impact of MR2938 in acute DSS-induced murine colitis as a model of epithelial injury relevant to IBD. We found that administration of MR2938 at 100 mg/kg via p.o. alleviated pathological symptoms and colonic inflammation in DSS-induced mouse model, and attenuated colonic damage by upregulating tight junction-associated proteins Occludin, ZO-1, and claudin-1. These findings provide solid evidence for the therapeutic potential of quinazolinone alkaloids and promote their potential for the treatment of inflammatory diseases.

## Materials and methods

### Cell culture and treatment

Raw264.7 cells and Caco-2 cells were obtained from the Cell Bank of the Chinese Academy of Sciences (Shanghai, China) and cultured in high-glucose DMEM medium supplemented with 10% fetal bovine serum (FBS) at 37 ℃ in a 5% CO_2_ atmosphere. Raw264.7 cells were seeded and incubated in six-well plates (8 × 10^5^ cells/well) overnight, and then treated with different concentrations of MR2938 for 2 h. Then Raw264.7 cells were activated by lipopolysaccharide (LPS, 100 ng/mL, Sigma-Aldrich Co., Shanghai, CN) for 2 h or 6 h for transcription or protein level analysis.

Caco-2 cells were seeded and incubated in 24-well plates (1 × 10^5^ cells/well) for 48 h and treated with 10 μmol/L of MR2938 for 2 h. Then 100 ng/mL of LPS was added for 24 h to induce tight function disruption.

### Animals

Male C57BL/6 mice (22 − 24 g) were purchased from Beijing Vital River Laboratory Animal Technology Co., Ltd. Mice were kept under pathogen-free conditions at ambient temperature (22 ± 2 °C) in a 12 h light–dark cycle with free access to standard food and water. The experimental procedures were performed according to the guidelines approved by the Institutional Animal Care and Use Committee of Ocean University of China.

### In vivo* PK study of MR2938*

MR2938 was synthesized in-house following previously reported procedure (Lv et al. 2023), and confirmed by NMR and HPLC chromatography (purity > 95%). To detect the pharmacokinetic characters of MR2938 in the plasma, the SD rats were orally and intravenous (i.v.) administered with MR2938 at a dose of 10 mg/kg and 3 mg/kg, respectively. The vehicle consisted of 10% DMSO, 30% saline, and 60% PEG400. Plasma samples were collected at 5 min, 15 min, 30 min and 1 h, 2 h, 4 h, 6 h, 8 h, 12 h, and 24 h after administration. After analyzing the concentration of MR2938 via LC–MS, the values of C_max_, T_max_, T_1/2_, Cl, AUC_last_, and AUC_Inf_ were calculated (Hu et al. 2018). The bioavailability of MR2938 from the p.o. was calculated by comparing the area under the concentration–time curve (AUC_Inf_) for the topical dose and that for the i.v. dose.

### Induction of colitis

After 1 week of acclimation, the C57BL/6 mice were randomly divided into the following groups (*n* = 6/group): control group, model group (DSS), DSS + 5-aminosalicylic acid (5-ASA, positive group, 100 mg/kg), DSS + MR2938 (100 mg/kg) and DSS + MR2938 (50 mg/kg). Colitis was induced by administration of 3% DSS drinking water for 5 consecutive days. Water consumption was monitored daily. At the same time, the mice in each group were given the corresponding agents: 5-ASA (100 mg/kg) and MR2938 (50 mg/kg or 100 mg/kg). The dosages of 5-ASA and MR2938 were adopted based on previous reports and our pilot trial. MR2938 or 5-ASA was dissolved in a mixture of saline and DMSO (95:5) to provide the desired concentrations.

Changes in bodyweight were assessed daily over the 9-day experimental period. On the last day of the experiment, mice were sacrificed, and the entire colon was excised. Colon length was measured and gently washed with physiological saline. Then two pieces with 0.5 cm in length of the distal section were used for histological assessment and immunofluorescence staining. The remaining colon tissue samples were used to determine MPO activity and the concentrations of cytokines.

### Histology analysis

Distal colons were harvested, fixed in 4% paraformaldehyde, and embedded in paraffin. Five-μm-thick tissue sections were stained with hematoxylin and eosin (HE) for light microscopic examination using a DP73 light microscope (Olympus, Tokyo, Japan). Colonic mucosa damage score was assessed as previously described. To count colonic goblet cells, fixed colonic tissues were also stained in Alcian blue for 10–15 min and dehydrated with 100% alcohol and xylene, followed by image acquisition on a microscope.

### Analysis of inflammatory cytokines and MPO activity

The serum was obtained from the collected blood samples through centrifugation at 3500 g for 15 min at 4 °C. A portion of frozen colon samples was homogenized with RIPA lysis buffer (Solarbio, Beijing, China) to extract total proteins. The homogenate was centrifuged at 12,000 × g at 4 °C for 15 min. The protein contents were quantified using a bicinchoninic acid (BCA) protein assay kit (Solarbio, Beijing, China) according to the manufacturer’s instructions. The concentrations of proinflammatory cytokines IL-1β, IL-6, and TNF-α in the serum and colon tissue were measured by ELISA (Jingmei Biotech, China) according to the manufacturers’ recommendations. MPO activity was measured using a myeloperoxidase assay kit (Jingmei Biotech, China).

### RNA extraction and RT-qPCR

Total RNAs of colon tissues were extracted using tissue RNA kit (Omega Biotek, China) following the manufacturer’s protocol. cDNA was obtained from RNA samples using ReverTra Ace qPCR RT Master Mix with gDNA Remover (TOYOBO, Japan). Quantitative real-time PCR was performed by Applied Biosystems^®^ QuantStudio Q5 instrument (Thermo, USA) with SYBR qPCR Master Mix (Vazyme, China). The relative gene expression was measured and normalized to β-actin expression using 2^−ΔΔCt^ method. The PCR sequences are shown in Table S3.

### Immunofluorescence staining

For immunofluorescence staining, the frozen colon slides or cells were fixed in 4% paraformaldehyde, permeabilized with 0.1% Triton X-100, blocked non-specific proteins in 5% BSA/PBS for 1 h, and incubated overnight at 4 °C with Occludin, ZO-1 or p65 primary antibodies. The slides were then washed with phosphate buffer (PBS) and incubated with Dylight 488 labeling secondary antibody for 1 h at room temperature. Finally, the colon slides were stained with DAPI for 10 min, and then imaged and captured using ECHO microscope.

### Western blot analysis

Total proteins were extracted with RIPA lysis buffer. After determining the protein concentration by the BCA assay kit, an equal amount of protein was subjected to SDS-PAGE and electro-transferred to a PVDF membrane. The membrane was immune detected with specific antibodies overnight at 4 °C. Proteins were quantified by Image J software. The target antibodies against p65, phosphorylated p65, IκBα, p-IκBα, TLR4, NLRP3, caspase-1, IL-1β, and β-actin were purchased from Proteintech or Cell Signaling Technology.

### Statistical analysis

Data were presented as the mean ± standard deviation (SD) of three independent experiments. The differences between multiple groups were analyzed by one-way ANOVA, followed by Dunnett’s multiple comparison test using GraphPad Prime 8 (GraphPad Software, San Diego, CA, USA). A value of *P* < 0.05 or *P* < 0.01 was defined as statistically significant.

## Results

### MR2938 alleviates pathological symptoms and colonic inflammation in DSS-induced colitis mouse

**Fig. 2:**
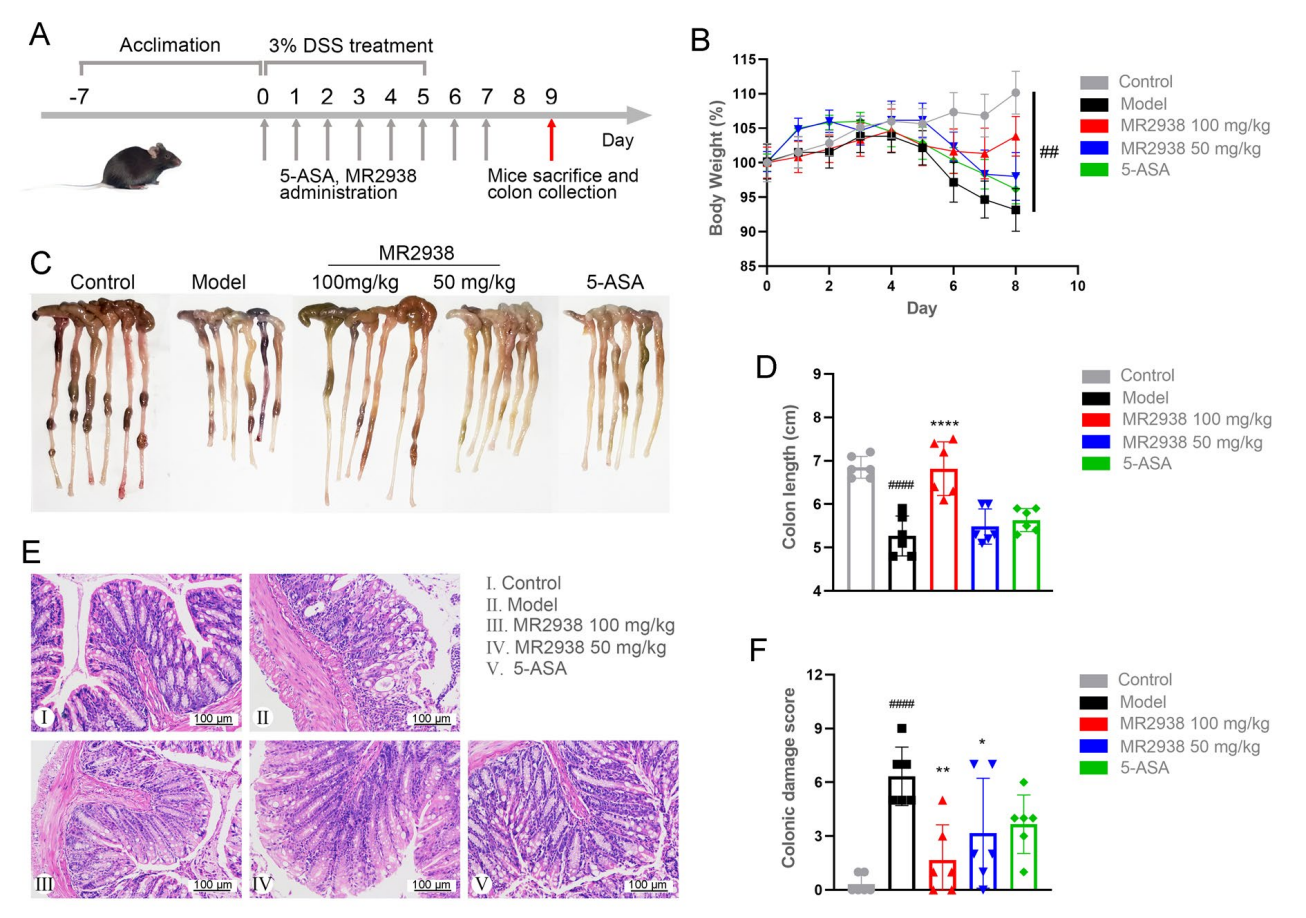
MR2938 alleviated the symptoms in DSS-induced colitis. **A** Experimental scheme for DSS-induced acute colitis and therapeutic administration of compounds. Mice were orally administered with PBS or MR2938 (100 or 50 mg/kg), or 100 mg/kg of 5-ASA. **B** Daily changes in body weight in different groups. **C** Macroscopic observation of colon and **D** the lengths of colon from each group. **E** Representative images of hematoxylin and eosin (HE)-stained colon tissue and **F** colonic damage score. Scale bar = 200 µm. Data are presented as the mean ± SD (*n* = 6). Statistical significance was determined using one-way ANOVA with Tukey tests for multiple-group comparisons. ^##^*p* < 0.01, ^####^*p* < 0.0001 compared with control group. *p < 0.05, **p < 0.01 and ****p < 0.0001 compared with model group

In the previous study, we observed that MR2938 has anti-inflammatory effects in BV2 and Raw264.7 cell lines. So, we first confirmed its anti-inflammatory efficacy in vivo in an acute DSS-induced murine colitis as a model of epithelial injury relevant to IBD. C57BL/6 mice were given 3% DSS in drinking water for 5 days, and two doses (50 mg/kg and 100 mg/kg) of MR2938 were administered via oral gavage for 7 days (Fig. 2A). Murine DSS-induced colitis could efficiently simulate the typical clinical phenotypes of IBD, such as weight loss, hematochezia, and diarrhea. Treatment of mice with different doses of MR2938 or 5-ASA (positive control) showed a marked improvement in body weight (Fig. 2B). Shortened colon length, a typical feature of colitis, was observed in the DSS-induced model group. And it was greatly relieved by the administration of 100 mg/kg of MR2938, similar to the control group, significantly longer than the 5-ASA group (Fig. 2C, D). Histological analysis further demonstrated that oral administration of MR2938 contributed to obvious attenuation of inflammatory cell infiltration, mucosal damage, and overall colonic damage score (Fig. 2E, F). In addition, organ indexes of mice displayed no obvious differences between the treatment group and control groups (Supporting Information Fig. S1). These studies suggested that MR2938 was in vivo efficacious for colitis with few toxic effects.

**Fig. 3:**
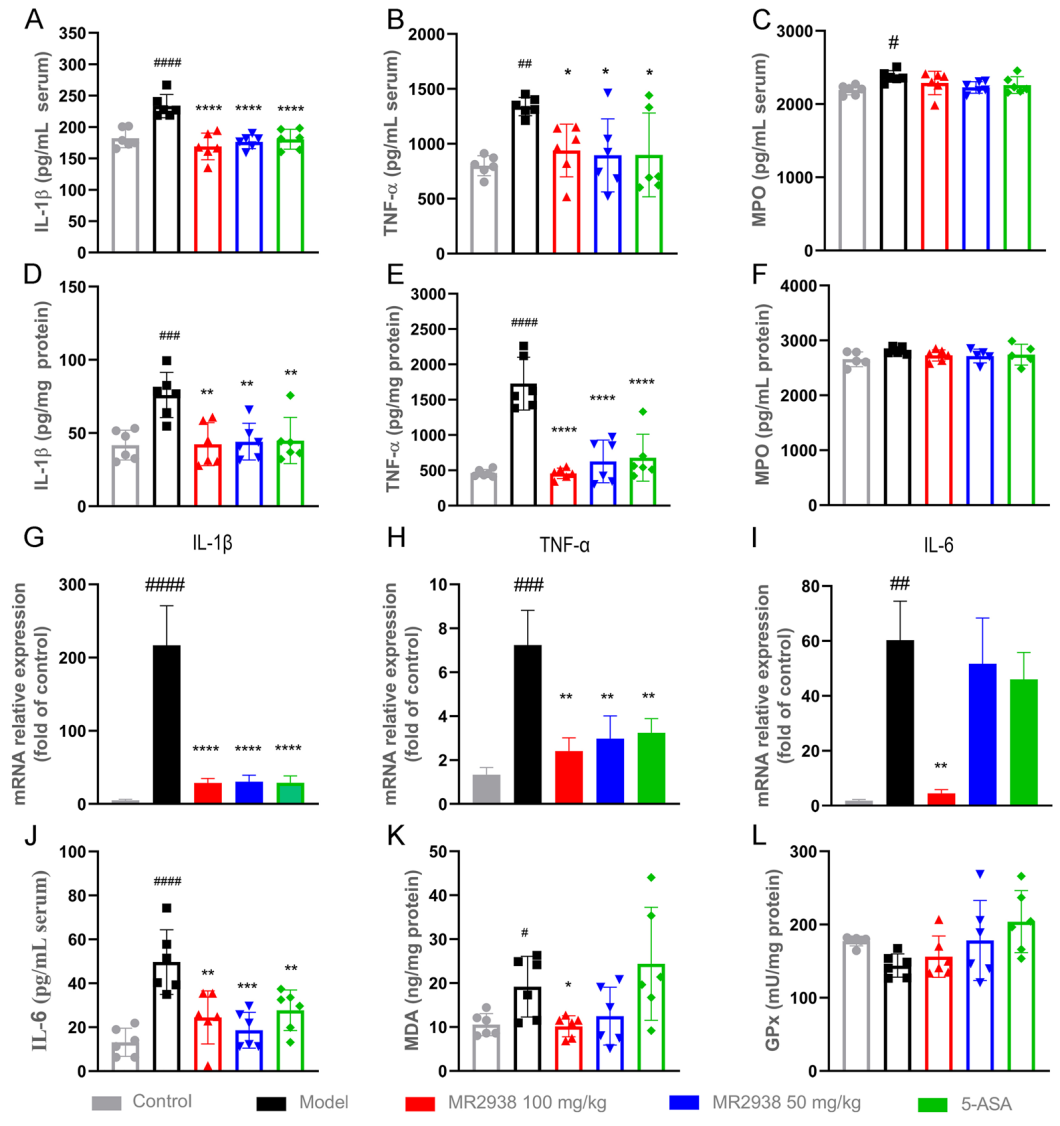
MR2938 suppressed inflammation response in colitis mice. Control or DSS-colitis mice were orally administered with PBS or MR2938 (100 or 50 mg/kg) or ASA (100 mg/kg). Concentrations of IL-1β (**A**), TNF-α (**B**) MPO (**C**), and IL-6 (**J**) in the serum. Concentrations of IL-1β (**D**), TNF-α (**E**), and MPO (**F**) in the colon. mRNA level of IL-1β (**G**), TNF-α (**H**), IL-6 (**I**) in the colon. Concentration of MDA (**K**) and GPx (**L**) in the colon. Data were presented as means ± SD (*n* = 6). Statistical significance was determined using one-way ANOVA, followed by Tukey test. ^#^*p* < 0.05, ^##^*p* < 0.01, ^###^*p* < 0.001, ^####^*p* < 0.0001 compared with control group. *p < 0.05, **p < 0.01 and ****p < 0.0001 compared with model group

Colitis can also result in increased production of proinflammatory cytokines and myeloperoxidase (MPO), a lysosomal protein in neutrophils, which is a key indicator of neutrophil infiltration into colonic tissues during intestinal inflammation. To further evaluate the impact of MR2938 on systemic and intestinal inflammatory responses, MPO and proinflammatory cytokines in the serum and colonic tissues were measured. DSS stimulation caused a marked increase in the production of IL-1β (Fig. 3A) and TNF-α (Fig. 3B) in mouse serum, while a significant decrease in these inflammatory molecules was observed in both MR2938 treatment groups dosing 50 mg/kg and 100 mg/kg. Similarly, MR2938 decreased the colonic levels of IL-1β (Fig. 3D) and TNF-α (Fig. 3E). Moreover, MR2938 treatment also resulted in a slight decrease of the concentration of MPO induced by DSS in serum and colon of mice (Fig. 3C, 3F). We then evaluated the mRNA level of inflammatory factors in colon. As expected, MR2938 noticeably inhibited the gene transcription of IL-1β (Fig. 3G) and TNF-α (Fig. 3H). Impressively, 100 mg/kg of MR2938 exhibited more favorable effects on the transcription of IL-6 than 5-ASA (Fig. 3I). We then used ELISA to analyze IL-6 levels in the serum of mice in each group. Although the serum IL-6 level was lower in the 50 mg/kg of MR2938 group compared with the 100 mg/kg of MR2938 group, there was no statistical difference between the two groups (Fig. 3J). The anti-oxidant stress effects of MR2938 were then measured, as exemplified by malondialdehyde (MDA) and glutathione peroxidase (GPx) in colon. MDA is one of the most important products of membrane lipid peroxidation, and its production can also aggravate membrane damage. GPx primarily scavenges lipid hydroperoxides by catalyzing the oxidation of GSH into GSSG. In our experiment, DSS significantly increased the level of MDA compared with control group, and MR2938 significantly suppressed the production of MDA (Fig. 3K). However, no significant difference in GPx was observed between colitis group and treatment group (Fig. 3L).

### MR2938 restores gut-barrier function in DSS-induced colitis

**Fig. 4:**
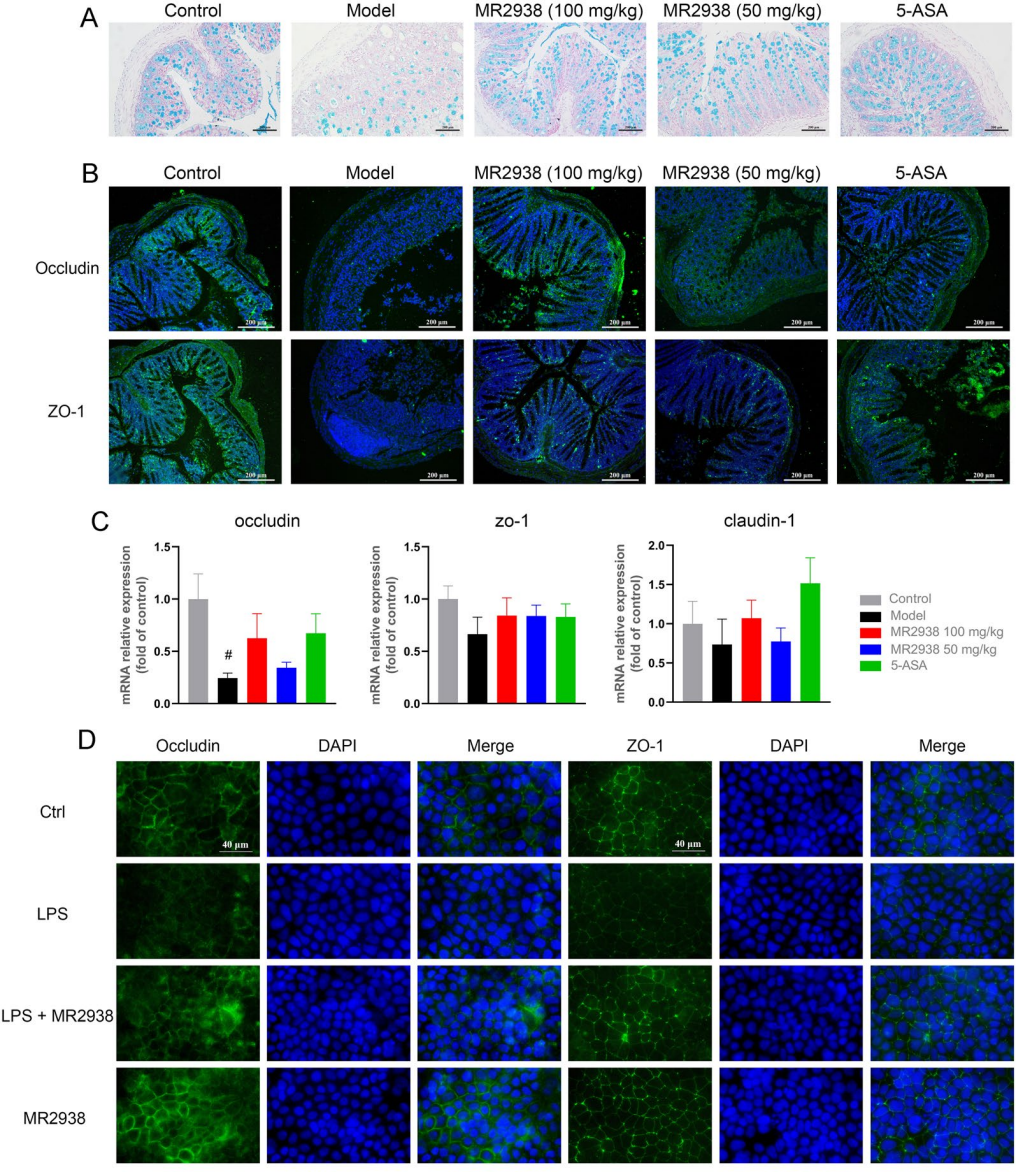
MR2938 attenuated colonic damage by upregulating tight junctions. **A** Representative images of Alcian blue-stained inner mucus layer of colonic sections. Scale bars = 200 μm. **B** Representative immunofluorescence images showing in situ expression of Occludin and ZO-1. Scale bar = 200 µm. **C** Occludin, ZO-1, and claudin-1 mRNA levels in colon tissue. **D** Representative immunofluorescence images expression of Occludin and ZO-1 in Caco-2 cells. Cells were treated with 10 μmol/L of MR2938 and then stimulated by LPS. Scale bar = 20 µm. Data are presented as the mean ± SD (*n* = 6). Statistical significance was determined using one-way ANOVA with Tukey tests for multiple-group comparisons. ^#^*p* < 0.05

Then to validate the restoration of MR2938 on the colonic mucosal barrier, mucin-secreting goblet cells in the colonic epithelia were measured using Alcian blue staining. DSS significantly reduced the thickness of colonic epithelial mucosa, and MR2938 treatment rescued the differentiation of mucin-producing goblet cells similar to the control group (Fig. 4A). The levels of pivotal tight junction-associated proteins Occludin, ZO-1, and claudin-1 in gut homeostasis were then investigated. Immunofluorescence staining results illustrated that the Occludin and ZO-1 proteins in DSS-treated mouse colon disappeared largely, whereas MR2938 could partially alleviate the depletion in a dose-dependence manner, and 100 mg/kg of MR2938 was as effective as 5-ASA (Fig. 4B). The mRNA levels of Occludin, ZO-1 and claudin-1 were also upregulated in the 100 mg/kg of MR2938 group compared with the colitis group, though there was no statistical difference between the two groups (Fig. 4C).

To further evaluate the protective role of MR2938 on intestinal epithelial barrier, we performed a culture experiment of Caco-2 stimulated by LPS. The results showed that LPS decreased the expression of Occludin and ZO-1, and MR2938 at 10 μmol/L was able to rescue expression levels (Fig. 4D). Meanwhile, MR2938 alone (without LPS) made little changes on the protein levels (Fig. 4D). The results indicated that MR2938 maintained the proper epithelium barrier by increasing the tight junctions without obvious toxicity.

### MR2938 suppressed LPS-induced inflammation through NF-kB signaling in macrophage

To explore the molecular regulatory mechanisms of MR2938 against cellular inflammation, we stimulated macrophage Raw264.7 cell line with LPS. NF-κB induces the expression of various proinflammatory genes, including those encoding cytokines and chemokines, and also participates in inflammasome regulation. We first examined the influence of MR2938 on IκBα and NF-κB subunit p65 (Fig. S2A-C). The phosphorylation of IkBα and p65 was significantly increased after LPS stimulation, whereas they were effectively inhibited by MR2938 treatment in a dose-dependent manner (Fig. S2A, B and Fig. S3). We also detected the levels of TLR4, one of the pattern-recognition receptors, in LPS-induced macrophages. Although the expression of TLR4 was reduced after MR2938, the decrease of TLR4 did not correlate to MR2938 concentration. In addition, it was observed that total p65 was slightly downregulated under MR2938 treatment. To confirm the effects of MR2938 on NF-κB, the subcellular location of NF-κB using immunofluorescence was checked (Fig. S2C). Following LPS stimulation, NF-κB fluorescence intensity was raised in the nucleus, suggesting that NF-κB quickly increased and phosphorylated into the nucleus. Following MR2938 treatment, we observed that NF-κB fluorescence intensity dropped and was mostly accumulated in the cytoplasm, suggesting that MR2938 inhibited NF-κB phosphorylation and had a slight impact on NF-κB expression. Then the expressions of IL-1β, NLRP3, and caspase-1, the target genes of NF-κB, were tested by qPCR and western blot (Fig. S2D-E). As we expected, both in mRNA and protein level, the expressions of IL-1β, NLRP3, and caspase-1 were significantly reduced with MR2938 treatment compared with those stimulated by LPS. Subsequently, to validate if the NLRP3 decrease was caused by NF-κB, an IκBα inhibitor IKK16, which can block, suppresses the LPS-induced phosphorylation level of NF-κB p65 without affecting p65 protein itself. As expected, the elevated expression levels of NLRP3 by LPS were obviously decreased by IKK16, similar to MR2938 (Fig. S2F). Taken together, the above results demonstrated that MR2938 suppressed NF-κB p65 activation to reduce inflammation.

## Discussion

Herein, we evaluated the effects of MR2938 on inflammation levels and epithelial barrier function via a head-to-head in vivo evaluation in a DSS-induced colitis mouse model. Based on the histology results and cytokine levels in serum and colon tissue, MR2938 demonstrated effective anti-inflammatory efficacy in vivo. IBD patients have higher levels of LPS than healthy people (Lu et al. 2024). LPS is known to determine acute inflammatory reactions. Macrophages, the gatekeepers of intestinal immune homeostasis, were used to analyze the possible anti-inflammatory mechanism of MR2938. NF-κB serves as a central mediator in the induction of proinflammatory genes and plays a pivotal role in modulating both inflammatory and immune responses. Within the context of IBD, aberrant activation and dysregulation of NF-κB have been identified as significant contributors to the pathogenesis of these conditions, and inhibition of NF-κB has been associated with alleviation of IBD occurrence (Laurindo et al. 2023; Rogler et al. 1998). The NF-κB proteins are sequestered in the cytoplasm by IκB family members, of which IκBα is the most important. Upon activation, IκBα is phosphorylated, thereby triggering ubiquitin-dependent IκBα degradation in the proteasome, resulting in rapid and transient nuclear translocation of canonical NF-κB members. In this study, MR2938 blocked the NF-κB signaling by decreasing the phosphorylation of IκBα, which inhibited the production of inflammatory factors such as IL-1β. In addition, the expression of NLRP3 and caspase-1 was also suppressed by MR2938 in a dose-dependent manner. The results are consistent with those previously reported, in which some NLRP3 inhibitors downregulate NLRP3 expression by NF-κB signaling ( Akther et al. 2021; Zahid et al. 2019). NF-κB could be activated by multiple signals, such as pattern-recognition receptors, proinflammatory cytokine receptors, T-cell receptors, and B-cell receptors (Vallabhapurapu et al. 2009). Toll-like receptor 4 (TLR4) functions as a sensor mediating the crosstalk between the intestinal commensal microbiome and host immunity; TLR4 deficiency enhances susceptibility to DSS-induced colitis (Liu et al. 2022). In the present study, although MR2938 reduced TLR4 levels in LPS-induced macrophages, no direct correlation was observed between the concentration of MR2938 and TLR4 inhibition. Further experimentation is warranted to elucidate the upstream signaling pathways of NF-κB that were modulated by MR2938.

Intestinal barrier damage likely resulted in bacterial translocation (Kiely et al. 2018; Twardowska et al. 2022). Furthermore, the loss of the mucus layer leads to the adherence of microbiota to the epithelium, accelerating the process of bacterial translocation (Grootjans et al. 2013). MR2938 administration increased the number of goblet cells in DSS-induced colitis mice and the thickness of epithelial mucosa, thereby contributing to restoring intestinal homeostasis. The alteration of tight junctions comprised of Occludin, ZO-1, and claudin damaged the barrier function and epithelial integrity (Kuo et al. 2021, 2022). In the present study, we found MR2938 promoted beneficial tight junctions in DSS-induced colitis mice. To further confirm these results, we examined the effect of MR2938 on the epithelium barrier using Caco-2 cells. Our observations revealed a significant reduction in the disruptive effects of LPS on tight junctions in Caco-2 cells when treated with MR2938. Collectively, these outcomes provided compelling evidence that MR2938 was beneficial to preserving the integrity of the intestinal epithelial barrier.

**Fig. 5:**
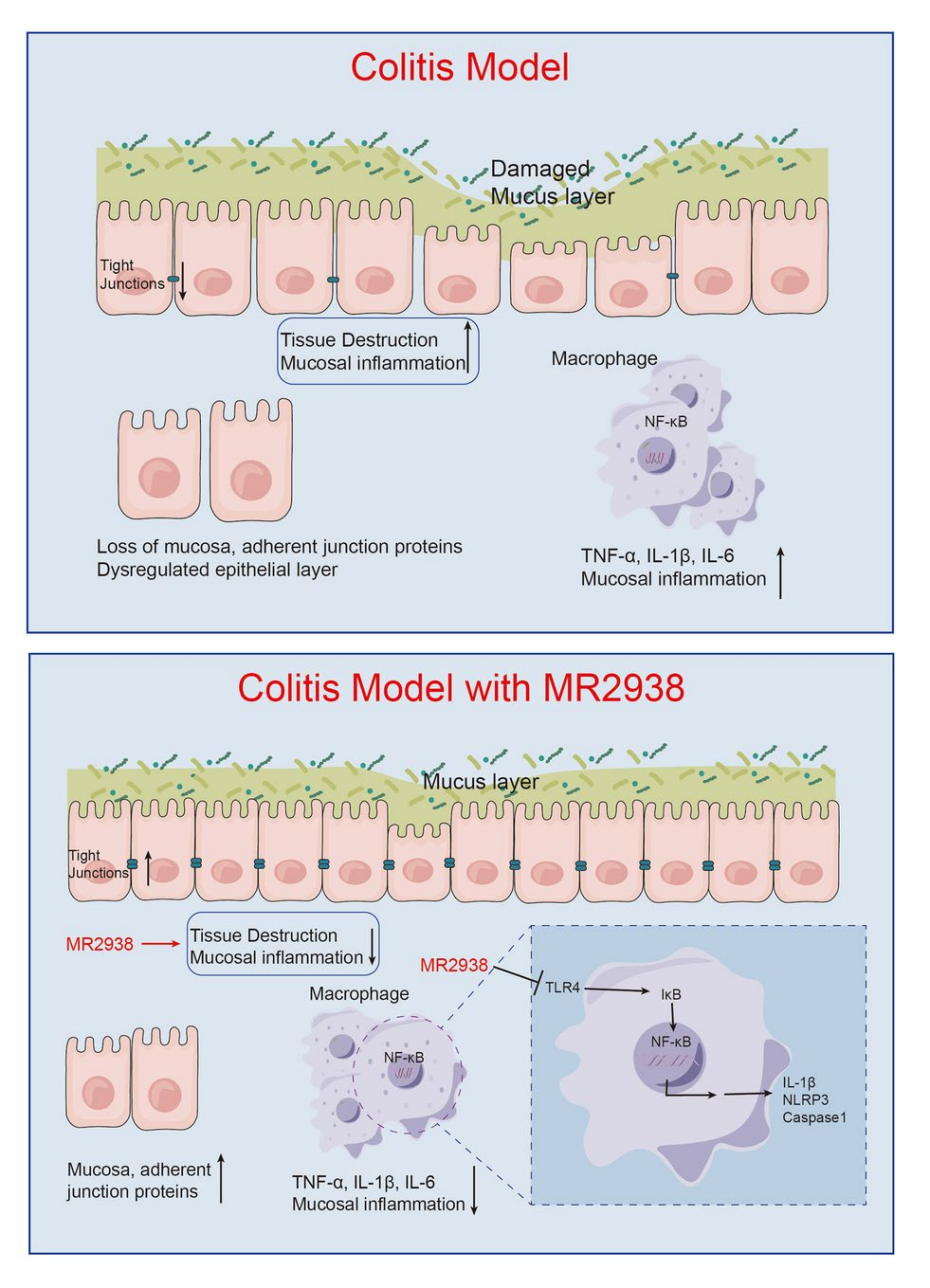
A schematic model showing the mechanism of MR2938 alleviated DSS-induced colitis. MR2938 attenuated colonic damage by upregulating tight junctions and inhibited the activation of intestinal immune cells, such as macrophages, by blocking NF-κB signaling to improve the integrity of the gut barrier and inhibit the inflammatory process in mice with colitis

Taken together, our study illustrated that MR2938 alleviated DSS-induced inflammatory responses and improved the intestinal barrier (Fig. 5)

. Thus, MR2938 is a promising anti-inflammatory lead compound worthy of more intensive preclinical investigations for the development of an oral therapeutic for IBD.

## Supplementary Information

Below is the link to the electronic supplementary material.

Supplementary file1 (DOCX 864 KB)

## Data Availability

The data that supports the findings of this study are included in this published article and its supplementary information file.
