# Supplementary material for: MR2938 relieves DSS-induced colitis in mice through inhibiting NF-κB signaling and improving epithelial barrier
Source: Mar Life Sci Technol. 2025 Mar 17;7(4):915–24. doi: 10.1007/s42995-025-00285-x (PMC12662929; doi:10.1007/s42995-025-00285-x)
Supplement: Supplementary file 1 — Supplementary file1 (DOCX 864 KB) [file 42995_2025_285_MOESM1_ESM.docx]

**Supporting information**

**MR2938 relieves DSS-induced colitis in mice through inhibiting NF-κB signaling and improving epithelial barrier**

Ling Lv^1,2^, Mireguli Maimaitiming^1,2^, Shuli Xia^1,2^, Jichen Yang^1,2^, Tiantian Zhang^1^, Yuming Wang^1^, Xin Li^1,2^, Iryna Pinchuk^3^, Pingyuan Wang^1,2^, Chang-Yun Wang^1,2^, Zhiqing Liu^1,2*^

^1^Key Laboratory of Marine Drugs and Key Laboratory of Evolution and Marine Biodiversity (Ministry of Education), School of Medicine and Pharmacy, Institute of Evolution & Marine Biodiversity, College of Food Science and Engineering, Ocean University of China, Qingdao 266003, China

^2^Laboratory for Marine Drugs and Bioproducts, Qingdao Marine Science and Technology Center, Qingdao 266237, China

^3^Division of Gastroenterology, Department of Medicine, Pennsylvania State Milton S. Hershey Medical Center, Hershey, Pennsylvania, 17033, USA

*Corresponding author: [liuzhiqing@ouc.edu.cn](mailto:liuzhiqing@ouc.edu.cn).

**Table of Contents**

**Table S1**. PK profiles of MR2938 in rat after administrations

**Table S2.** Concentration of MR2938 in brain after its i.p. administration

**Table S3**. Primer sequences for qRT-PCR analysis.

**Figure S1**. The organ weight and liver function index in DSS-induced colitis.

**Figure S2**. MR2938 inhibited NF-κB activation *in vitro*.

**Figure S3**. Quantitative Western Blot analysis of p-p65/ p65 levels in Raw264.7 cells.

**Figure S4**. ^1^H NMR spectra of **MR2938**.

**Figure S5.** ^13^C NMR spectra of **MR2938**

**Figure S6.** HPLC chromatograms of **MR2938**

**Table S1**. PK profiles of MR2938 in rat after administrations ^a^

| **PK parameter** | **T_1/2_ (h)** | **T_max_ (h)** | **C_max_ (ng/mL)** | **AUC_last_ (h·ng/mL)** | **AUC_Inf_ (h·ng/mL)** | **Cl (mL/min/kg)** | ***F* (%)** |
| --- | --- | --- | --- | --- | --- | --- | --- |
| **3 mg/kg i.v.** | 2.25 | - | 1,026 | 1,059 | 1,071 | 47.2 | - |
| **10 mg/kg p.o.** | 2.87 | 0.667 | 592 | 2,075 | 2,107 | - | 59 |

^a^Expressed as mean, n = 3. T_1/2_, half-life; T_max_, time for reaching C_max_; C_max_, maximum drug concentration; AUC, area under the curve; Cl, plasma clearance; *F*, bioavailability

**Table S2**. Concentration of MR2938 in brain after its i.p. administration (6.5 mg/kg)

| **Parameter** | **Value of MR2938 (n=3)** |
| --- | --- |
| Concentration (ng/g) | 222.07 |
| Amount (ng) | 236.7 |
| i.p. administration (μg) | 145 |

**Table S3**. Primer sequences for qRT-PCR analysis.

| Gene | Forward Primer Sequence | Reverse Primer Sequence |
| --- | --- | --- |
| β-actin | GACGTTGACATCCGTAAAGAC | CCACCGATCCACACAGAGTA |
| IL-6 | TGGAGTCACAGAAGGAGTGGCTAAG | TCTGACCACAGTGAGGAATGTCCAC |
| IL-1β | AGACAACTGCACTACAGGCTC | GTGGGTGTGCCGTCTTTCAT |
| TNF-α | ACCACGCTCTTCTGTCTACT | GGCTACAGGCTTGTCACTC |
| NLRP3 | TCCCAGACACTCATGTTGCC | GTCCAGTTCAGTGAGGCTCC |
| caspase-1 | ACTGACTGGGACCCTCAAGT | GCAAGACGTGTACGAGTGGT |
| ZO-1 | GCAGACCCAGCAAAGGTGTA | GTATGACGGCTGCTCAAGGT |
| occludin | GGTTGATCCCCAGGAGGCTA | CCGATCCATCTTTCTTCGGGT |
| Claudin-1 | CAACCCGAGCCTTGATGGTA | ACTAATGTCGCCAGACCTGAAA |


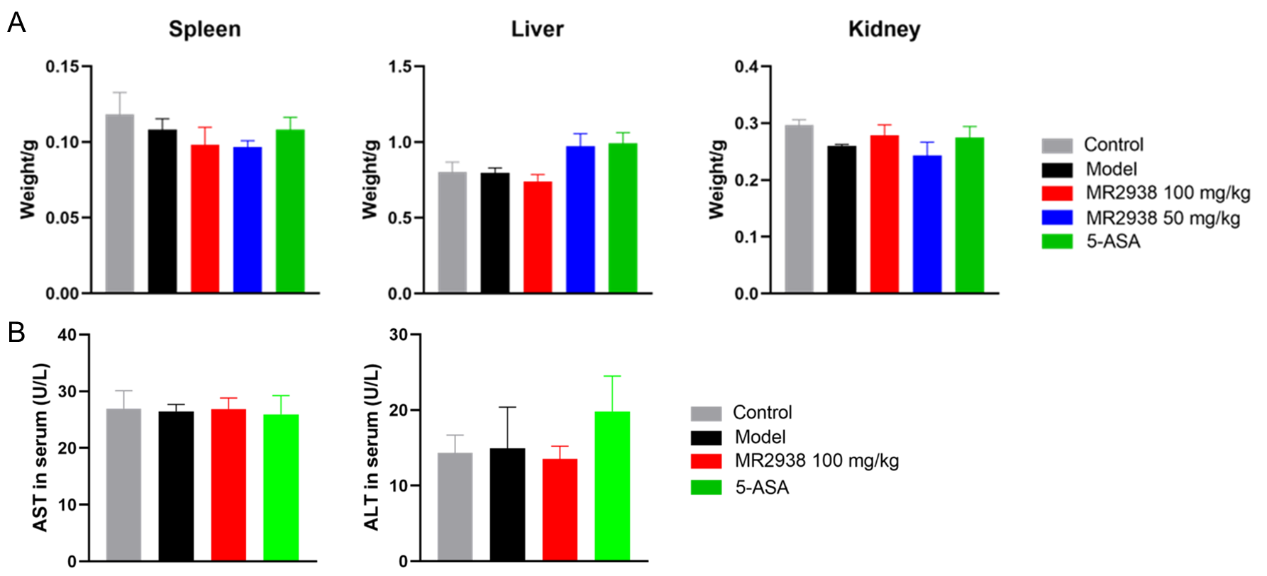


**Figure S1**. The organ weight and liver function index in DSS-induced colitis. (A) The weight of spleen, liver and kidney in different groups. (B) The AST and ALT activity in serum.


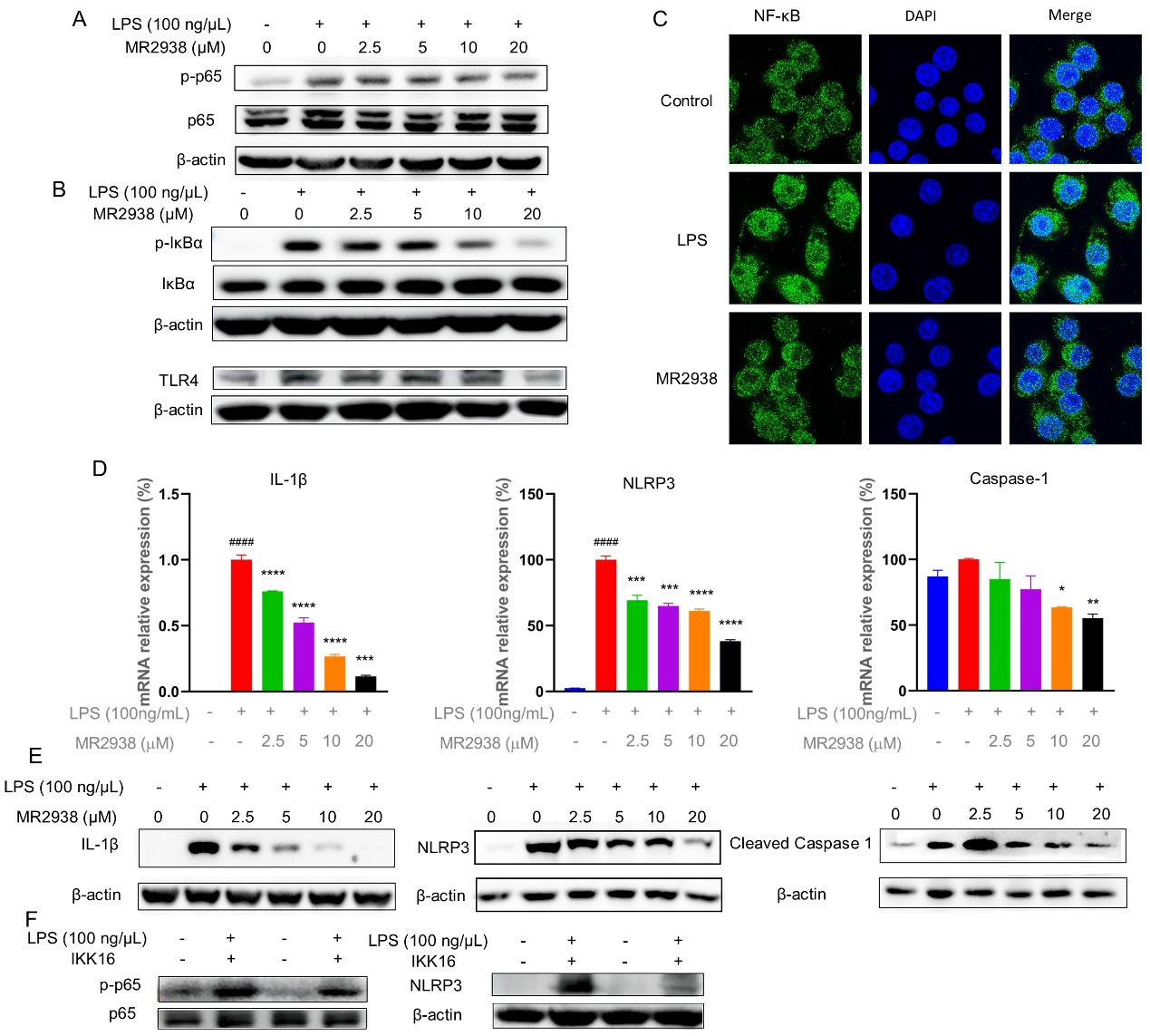
 **Figure S2.** MR2938 inhibited NF-κB activation *in vitro*. Raw264.7 cells were treated with different concentration of MR2938 and stimulated by LPS. (A-B) The protein levels of p65, p-p65, IκB, p-IκB, and TLR4 analyzed by western blot in Raw264.7 cells. (C) Fluorescent microscopy image analysis of NF-κB in Raw264.7 cells with 10 μM of MR2938 pre-treatment after LPS activation. (D-E) Effects of IL-1β, NLRP3 and caspase-1 expression by (C) qPCR and (D) western blot (E). (F) The protein levels of p65, p-p65 and NLRP3 in Raw264.7 cells with IKK inhibitors to block the NF-κB pathway. ^####^p < 0.0001 compared with control group. *p < 0.05, **p < 0.01, ***p < 0.001 and ****p < 0.0001 compared with model group.


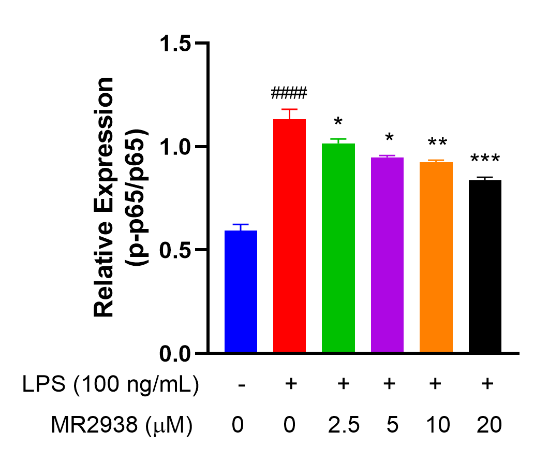


**Figure S3**. Quantitative Western Blot analysis of p-p65/ p65 levels in Raw264.7 cells.


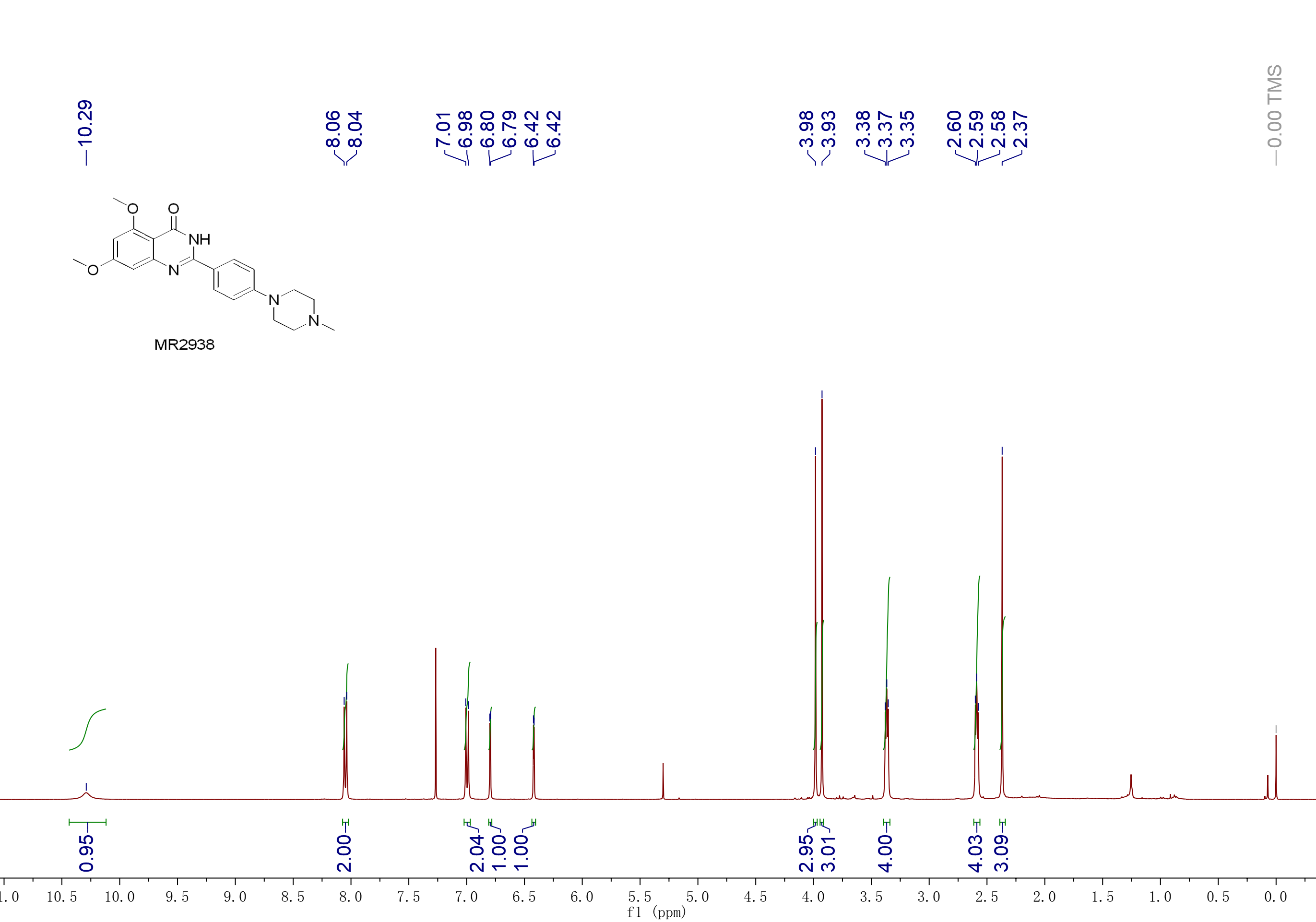


**Figure S4**. The copy of ^1^H NMR spectrum (400 MHz, CDCl_3_) of **MR2938**.


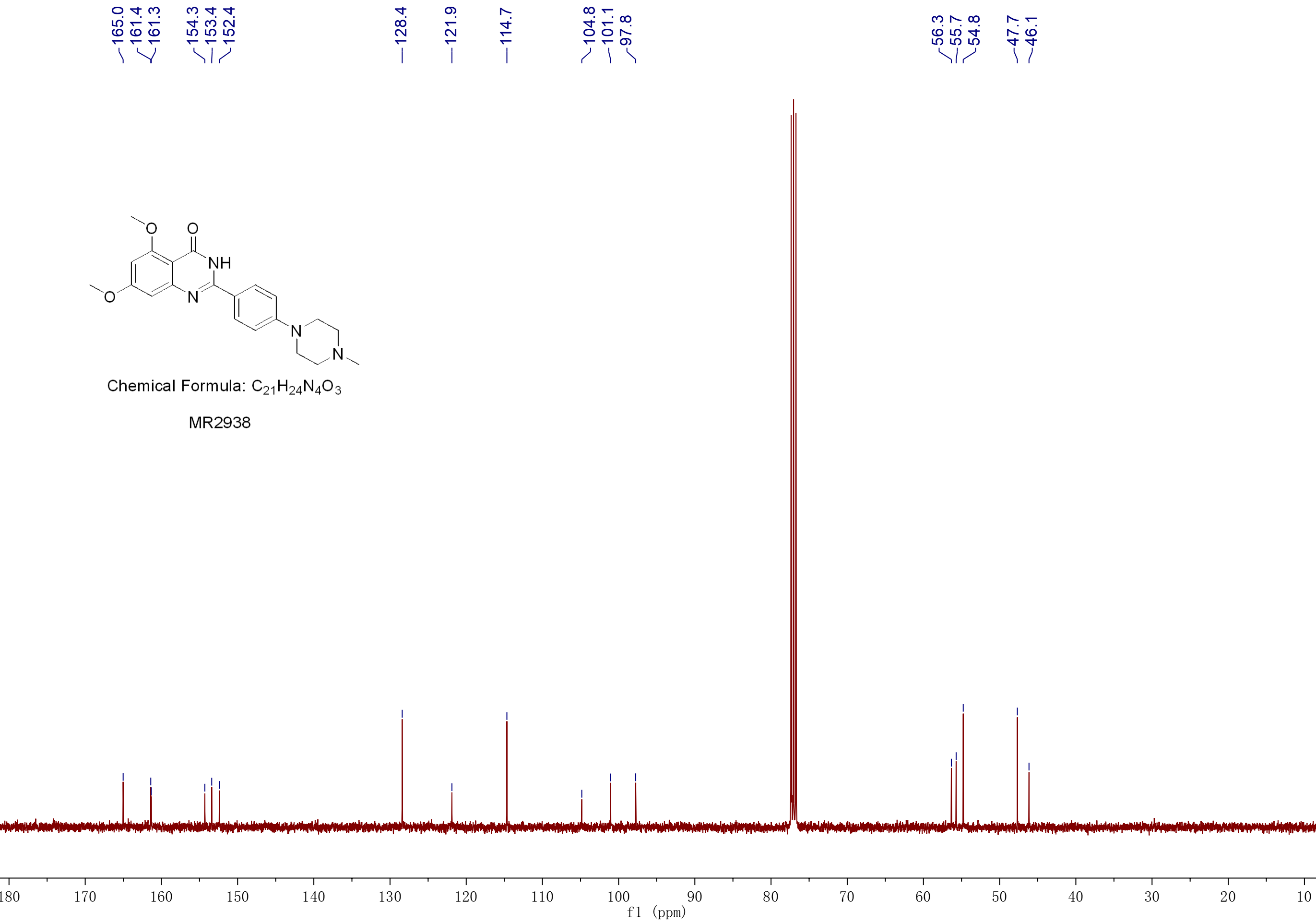


**Figure S5**. The copy of ^13^C NMR spectrum (100 MHz, CDCl_3_) of **MR2938**


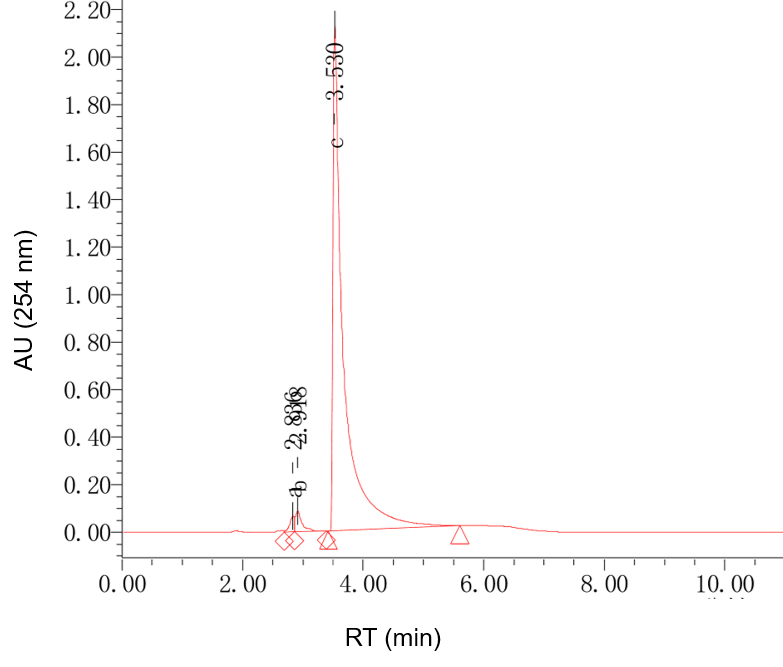


| Peak | RT (min) | Peak area (AU*min) | % area |
| --- | --- | --- | --- |
| a | 2.836 | 357446 | 1.31 |
| b | 2.918 | 684050 | 2.51 |
| c | 3.530 | 26161661 | 96.17 |

**Figure S6**. HPLC profile of MR2938
